# Impact of unplanned second debridement, antibiotics and implant retention on long‐term outcomes in knee exchange arthroplasty: Elevated risk of failure and reinfection

**DOI:** 10.1002/jeo2.12024

**Published:** 2024-04-30

**Authors:** Yu‐Chih Lin, Wei‐Cheng Chen, Shih‐Hui Peng, Chih‐Hsiang Chang, Sheng‐Hsun Lee, Sheng‐Hsuan Lin

**Affiliations:** ^1^ Department of Orthopaedic Surgery Chang Gung Memorial Hospital (CGMH) Kweishan Taoyuan Taiwan; ^2^ Bone and Joint Research Center Chang Gung Memorial Hospital (CGMH) Kweishan Taoyuan Taiwan; ^3^ College of Medicine Chang Gung University (CGU) Kweishan Taoyuan Taiwan; ^4^ Institute of Statistics National Yang Ming Chiao Tung University Hsinchu Taiwan

**Keywords:** debridement, antibiotics and implant retention (DAIR), implant failure, periprosthetic infection, reinfection, staged exchange arthroplasty

## Abstract

**Purpose:**

This study investigates the outcomes of two‐stage exchange arthroplasty (EA) for periprosthetic joint infection (PJI) following initial or unplanned repeat debridement antibiotics, and implant retention (DAIR).

**Methods:**

We retrospectively reviewed cases of knee arthroplasty infection treated with two‐stage EA after DAIR, spanning from January 1994 to December 2010. A total of 138 patients were included, comprising 112 with initial DAIR and 26 with an unplanned second DAIR. Data on demographics, comorbidities, infection characteristics and causative organisms were analyzed. The primary outcome was implant failure or reinfection, observed over a minimum follow‐up of 10 years.

**Results:**

The overall success rate for two‐stage EA was 87% (119/138 patients). Factors identified for treatment failure included reinfection with the same pathogen for unplanned second DAIR (hazard ratio [HR] = 3.41; 95% confidence interval [CI] = 1.35–4.38; *p* = 0.004), higher reinfection rates in patients undergoing EA after an unplanned second DAIR, especially with a prior history of PJI within 2 years (HR = 4.23; 95% CI = 2.39–5.31; *p* = 0.002), pre‐first DAIR C‐reactive protein (CRP) levels over 100 mg/dL (HR = 2.52; 95% CI = 1.98–3.42; *p* = 0.003) and recurrence with the same pathogen (HR = 2.35; 95% CI = 1.32–4.24; *p* = 0.007). Additional factors such as male gender (HR = 3.92; 95% CI = 1.21–5.25; *p *= 0.007) and osteoporosis (T score < −2.5; HR = 3.27; 95% CI = 1.23–5.28; *p* = 0.005) were identified as risk factors for implant failure in all EA cases.

**Conclusions:**

This study identifies key risk factors for worse knee EA outcomes following DAIR, including a pre‐first DAIR CRP level over 100 mg/L, same pathogen recurrence, and PJI history within 2 years. It shows implant failure rates remain constant across EA cases, regardless of DAIR sequence, particularly with risk factors like male gender and severe osteoporosis (T score < −2.5). These results underscore the need for careful evaluation before an unplanned second DAIR, given its significant impact on EA success.

**Level of Evidence:**

Level III.

**Abbreviations**
CI: confidence interval
CRP: C‐reactive protein
DAIR: debridement, antibiotics and implant retention
EA: exchange arthroplasty
ESR: erythrocyte sedimentation rate
HR: hazard ratio
MSIS: Musculoskeletal Infection Society
PJI: periprosthetic joint infection
SPSS: Statistical Package for the Social Sciences

## INTRODUCTION

Periprosthetic joint infection (PJI) is a prevalent and severe complication in total joint arthroplasty, leading to failure and significant patient morbidity [1, 14]. The incidence of PJI in knee arthroplasties ranges between 0.3% and 4% [3, 4], posing substantial challenges in orthopaedic practice. Treatment options for acute PJI include debridement, antibiotics and implant retention (DAIR), as well as one‐stage and two‐stage exchange arthroplasty (EA). While treatment success varies considerably based on multiple factors [23], the gold standard for chronic PJI has been two‐stage EA, boasting infection‐free survival rates of 80%–100% [12, 33]. Despite its effectiveness, two‐stage EA is associated with potential bone loss, soft tissue damage, and increased costs, often leading to poorer functional outcomes compared to DAIR [13]. Given these concerns, surgeons often initially opt for DAIR in acute cases to preserve the prosthesis and avoid more complex surgeries, despite its variable success rates of 58%–71% [20].

The 2018 International Consensus Meeting recommends two‐stage EA after an initial DAIR [2, 31], citing that success rates of subsequent unplanned second DAIRs, which vary between 50% and 74.3% as per literature, are generally lower [20, 29, 31, 34]. Our study uniquely defines ‘unplanned second DAIR’ as a procedure following a successful initial DAIR to critically assess its outcomes and influence on future two‐stage EAs, addressing a gap in current research.

Our study is structured with three primary objectives: (1) to ascertain the success rate of unplanned second DAIR in comparison to two‐stage exchange EA; (2) to contrast the outcomes of two‐stage EA in patients who have undergone either one DAIR or an unplanned second DAIR; and (3) to uncover clinical factors, encompassing both patient and surgical aspects, that are indicative of two‐stage EA failure in this patient group. Therefore, our research is predicated on the hypothesis that an unplanned second DAIR may significantly affect the success rates and overall outcomes of later two‐stage EA in the treatment of PJI. By focusing on these areas, our study aims to bridge the knowledge gap regarding the repercussions of repeated DAIR procedures and to enhance clinical decision‐making processes in the management of PJI cases.

## MATERIALS AND METHODS

PJI patients who were treated in our institute from January 1994 to December 2010 with institutional review board approval (IRB: 201601034B0) were collected in this retrospective cohort study. Informed consent was obtained from all individual participants included in the study.

All first PJIs following a primary total knee prosthesis in the aforementioned database with a follow‐up of ≥10 years were investigated and 11 patients who had unclear records or were not following the PJI treatment protocols of our institute were excluded. The study included a total of 699 patients, all of whom received a posterior‐stabilized design implant. Among these, 466 were treated with the Zimmer NexGen Complete Knee Solution LPS‐Flex, while the remaining 233 received the United U2 Knee System.

**Figure 1:**
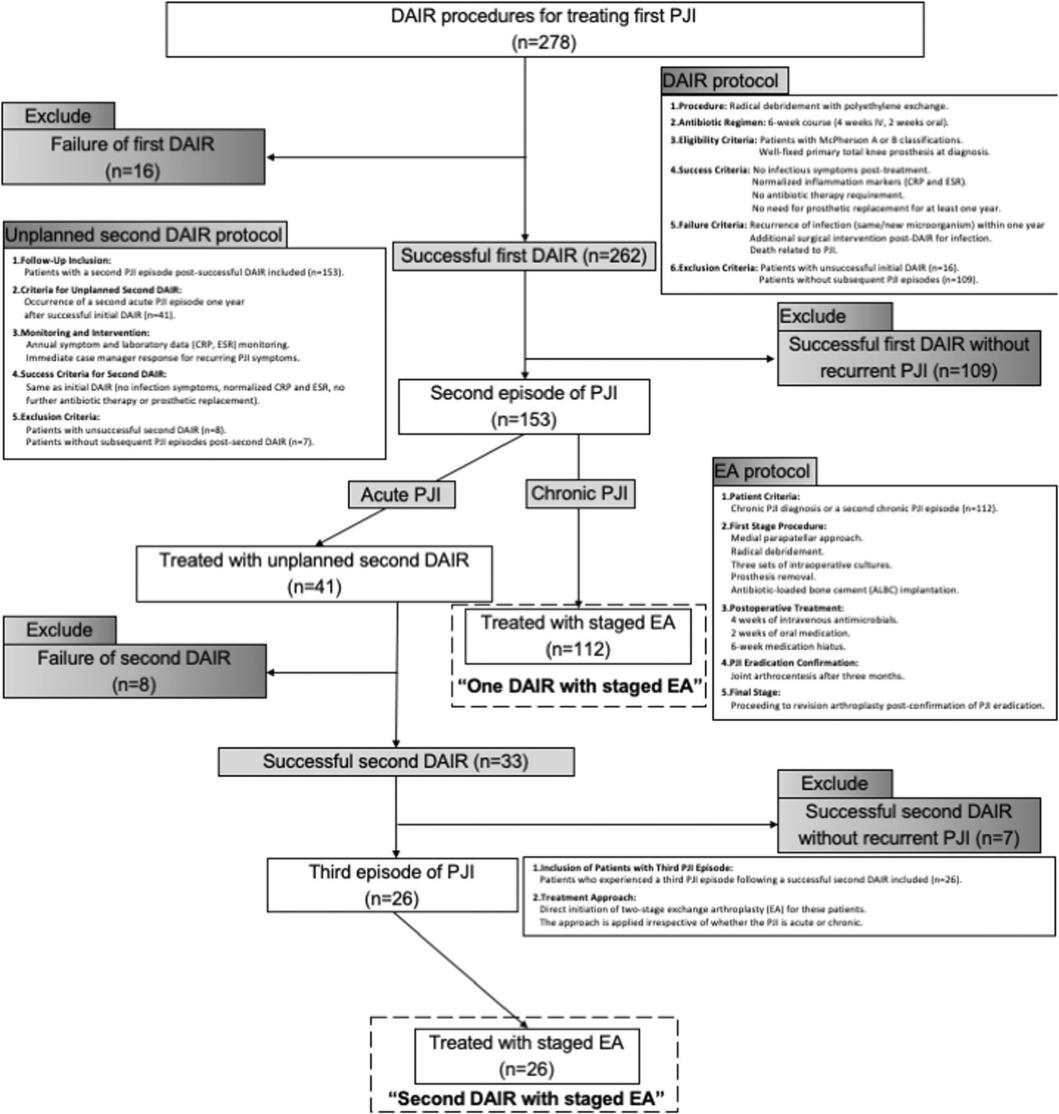
Flowchart of study selection and treatment protocol. Excluded cases are indicated by the grey background. DAIR, surgical debridement, antibiotics and implant retention; EA, exchange arthroplasty; PJI, periprosthetic joint infection.

In this study, we included 278 patients who underwent DAIR as the initial treatment for their first PJI. We then focused on analyzing these patients' clinical progression leading up to EA, as illustrated in Figure 1. Throughout the study period, which extended until 31 December 2020, continuous follow‐up for all participants was ensured by two dedicated case managers from our institute. We focused on the impact of unplanned second DAIR versus initial DAIR on the outcomes of subsequent two‐stage EA.

## PRIMARY AND SECONDARY ENDPOINTS

The primary outcomes evaluated in this study were the effectiveness of unplanned second DAIR and the results of two‐stage EA. We specifically focused on identifying risk factors associated with two key endpoints: reinfection and implant failure. Reinfection was defined as meeting the PJI criteria (before 2011, it was based on a positive culture from at least two separate tissue samples from the knee joint; after 2011, per Musculoskeletal Infection Society [MSIS] [24]) without reliance on antibiotic suppression therapy. Implant failure was characterized by the necessity for implant removal due to either loosening or the requirement for a repeat two‐stage EA. Additionally, we conducted a detailed analysis of risk factors specifically contributing to reinfection following an unplanned second DAIR.

The endpoint of the study was meticulously defined as either implant failure or reinfection, adhering to the PJI criteria post two‐stage EA. Implant loosening was determined radiographically, characterized by features such as radiolucent lines wider than 2 mm at the cement‐bone or metal‐cement interface, progressive translucency, component migration, or cement fractures [27].

For this retrospective study, patient data were meticulously collected, including joint fluid analysis, culture results, laboratory data, and histological findings. The definition of PJI evolved during the study period: before 2011, it was based on a positive culture from at least two separate tissue samples from the knee joint. Post 2011, PJI was defined according to the MSIS criteria [24], and patients were treated following our institute's established protocols [19]. Acute hematogenous PJI was categorized by the acute onset of PJI symptoms and signs lasting no longer than four weeks prior to DAIR [30]. In contrast, symptoms present for more than 4 weeks indicate a chronic infection, for which the preferred treatment is two‐stage EA [19, 25].

## TREATMENT PROTOCOL AND PATIENT INCLUSION FLOWCHART

### DAIR protocol

Our DAIR protocol entailed radical debridement with polyethylene exchange, followed by a comprehensive 6‐week antibiotic regimen (4 weeks intravenous, followed by 2 weeks oral). Eligibility for this protocol was limited to patients with McPherson A or B [8] classifications, who had a well‐fixed primary total knee prosthesis upon diagnosis confirmation. A DAIR was considered successful if the patient exhibited no infectious symptoms, normalized inflammation markers (C‐reactive protein [CRP] and erythrocyte sedimentation rate [ESR]), was off antibiotic therapy, and did not require prosthetic replacement for at least 1‐year post‐treatment. Conversely, a DAIR was deemed unsuccessful if, within 1 year, there was a recurrence of infection (same or new microorganism), any additional surgical intervention due to infection post‐DAIR, or death related to PJI. Patients with unsuccessful initial DAIR were excluded from this study (*n* = 16). Patients without further episodes of PJI were also excluded from this study (*n* = 109) (Figure 1).

### Follow‐up and second DAIR inclusion

During follow‐up, patients who experienced a second episode of PJI after a previously successful DAIR (*n* = 153) were included. An unplanned second DAIR was considered if a patient encountered a second episode of acute PJI 1 year after a successful initial DAIR (*n* = 41). Annual monitoring of symptoms and laboratory data (CRP and ESR) was conducted in the outpatient department, with immediate case manager intervention if PJI symptoms recurred. The criteria for a successful second DAIR mirrored those of the initial DAIR. Patients with unsuccessful second DAIR were also excluded (*n* = 8). Patients without further episodes of PJI were also excluded from this study (*n* = 7) (Figure 1).

### Two‐stage EA protocol

For patients diagnosed with chronic PJI or encountering a second episode of chronic PJI, two‐stage EA was performed following a stringent protocol (*n* = 112). The first stage involved medial parapatellar approach with radical debridement and three sets of intraoperative cultures, prosthesis removal, and antibiotic‐loaded bone cement implantation. Postoperative treatment included 4 weeks of intravenous antimicrobials followed by 2 weeks of oral medication. A 6‐week medication break ensued. After 3 months, joint arthrocentesis was conducted to confirm PJI eradication before proceeding to the final revision arthroplasty.

### Third PJI episode and EA inclusion

Patients experiencing a third PJI episode (*n* = 26) after a successful second DAIR were included. In these cases, two‐stage EA was directly initiated, irrespective of the acute or chronic nature of the PJI. The patient flowchart and inclusion criteria are summarized in Figure 1.

#### Covariates

Patient demographics, comorbidities (including diabetes with diabetic complications, congestive heart failure, peripheral vascular disease, chronic pulmonary disease, mild and severe liver disease, hemiplegia, renal disease, leukaemia, lymphoma, metastatic tumour and acquired immunodeficiency syndrome), arthroplasty characteristics, pre‐treatment laboratory tests and PJI procedures with causative pathogens were all recorded and analyzed.

#### Statistical analysis

Follow‐up time (755–6156 days) was defined as the time from the final stage EA (reimplant date for two‐stage EA) to the date of reinfection, subsequent revision surgery, death, or study end date (31 December 2020), whichever came first. We used analysis of variance to compare continuous variables if they showed a normal distribution and used Kruskal–Wallis test if not. To ascertain the appropriate sample size for our study, a detailed power analysis was performed using G*Power software. We aimed for a power of 0.80, which is standard in clinical research, to detect clinically significant differences between our study groups. The effect size was estimated based on previous similar studies in the field, and an alpha level of 0.05 was set for statistical significance. Based on these parameters, the power analysis suggested a minimum sample size of 25 patients, allowing us to confidently detect differences in outcomes such as reinfection rates and implant survival between patients undergoing initial DAIR (*n* = 112) and those requiring unplanned second DAIR (*n* = 26). We evaluated the relationship of qualitative variables using the chi‐square or Fisher's exact tests. The suspected risk factors contributing to treatment failure (reinfection or implant failure of two‐stage EA and reinfection of unplanned second DAIR) were evaluated using univariate Cox regression models. Covariates identified from the univariate Cox regression analysis were included in the multivariable analysis to identify potential risk factors. For our study, further multivariate analysis was conducted only for variables with a significance level of less than 0.1 in the initial analysis. To validate our results, we employed specific multiple comparison tests, including the Bonferroni correction and Tukey's honestly significant difference test, to rigorously assess the impact of potential confounding factors and ensure the reliability of our findings. Survival analysis, taking into account time to reinfection and time to revision, was performed using Kaplan–Meier survival analysis. For all tests, *p* < 0.05 was considered significant. Processing and data analysis were performed using IBM Statistical Package for the Social Sciences (SPSS) Statistics for Windows, version 20.0 (IBM Corp.).

## RESULTS

### Comparison of demographic characteristics in EA groups post‐DAIR

We compared the demographic characteristics of patients who underwent two‐stage EA after either one successful DAIR or an unplanned successful second DAIR. Our analysis revealed no significant differences between these groups (Table 1). The overall success rate for unplanned second DAIR was 80.6% (33/41), while for EA, it was 87.0% (119/138). Specifically, the success rate for EA after the first DAIR was 90.2% (101/112), compared to 69.2% (18/26) after an unplanned second DAIR.

**Table 1:** Demographic characteristics between patients who underwent staged exchange arthroplasty with previous first DAIR and second DAIR.

Variables	First DAIR (112)	Unplanned Second DAIR (26)	*p*
Basic data			
Male/female	72 (64.3%)/40 (35.7%)	11 (42.3%)/15 (57.7%)	n.s.
Age (medium) (range) (IQR) (SD)	61.2 (63.5) (19.6–87.9) (21.5) (14.4)	64.2 (68.6) (35.3–84.0) (21.0) (14.2)	n.s.
Body mass index (medium) (range)	26.8 (26.1) (15.2–47.9)	26.5 (26.6) (18.5–34.6)	n.s.
(IQR) (SD)	(5.85) (5.68)	(4.92) (4.01)	
Albumin level (SD)	3.75 (0.560)	3.26 (0.756)	n.s.
eGFR (SD)	68.8 (33.5)	56.2 (30.4)	n.s.
CRP (SD)	53.4 (50.3)	53.7 (71.5)	n.s.
Underlying disease			
Charlson comorbidity index			
0	5 (4.5%)	0 (0%)	n.s.
1	14 (12.5%)	3 (11.5%)	
2	19 (17.0%)	3 (11.5%)	
3	20 (17.9%)	5 (19.2%)	
4	23 (20.5%)	5 (19.2%)	
5	18 (16.1%)	3 (11.5%)	
6	3 (2.7%)	6 (23.1%)	
7	4 (3.6%)	0 (0%)	
8	2 (1.8%)	0 (0%)	
McPherson type A	74 (66.1%)	18 (69.2%)	n.s.
McPherson type B	38 (33.9%)	8 (30.8%)	n.s.
Hypertension (%)	8 (7.1%)	2 (7.7%)	n.s.
Diabetes (%)	40 (35.7%)	12 (42.6%)	n.s.
Liver cirrhosis (%)	0 (0%)	0 (0%)	n.s.
COPD (%)	8 (7.1%)	2 (7.7%)	n.s
Rheumatic arthritis (%)	0 (0%)	0 (0%)	n.s.
Renal insufficiency (%)	8 (7.1%)	2 (7.7%)	n.s.
Osteoporosis with T score < −2.5 (%)	24 (21.4%)	6 (23.1%)	n.s.
Smoking habit (%)	10 (8.9%)	3 (11.5%)	n.s.
Use of immunosuppressive drugs (%)			
<2.5 mg Prednisolone/day (ever used)	0 (0%)	1 (3.8%)	n.s.
≥2.5 mg Prednisolone/day (ever used)	0 (0%)	1 (3.8%)	n.s.

Abbreviations: CI, confidence interval; COPD, chronic obstructive pulmonary disease; CRP, C‐reactive protein; DAIR, surgical debridement, antibiotics and implant retention; eGFR, estimated glomerular filtration rate; IQR, interquartile range; SD, standard deviation.

### Surgical and bacterial factors affecting outcomes

The groups requiring three‐stage EA (16.1% vs. 38.5%; *p* = 0.043) and four‐stage EA (1.8% vs. 7.7%; *p* = 0.012) differed significantly between one DAIR before EA and second DAIR before EA. A notable difference was also observed in the history of subsequent PJI within 2 years (6.23% vs. 26.1%; *p* = 0.024). Additionally, the prevalence of cultures showing the same species between the last DAIR, and two‐stage EA was significantly lower in the first DAIR group compared to the second (24.1% vs. 30.8%; *p* = 0.025) (Table 2).

**Table 2:** Surgery‐related and bacterial characteristics of patients who underwent staged exchange arthroplasty after first or second DAIR.

Variables	First DAIR (112)	Unplanned second DAIR (26)	*p*
Surgery‐related factors			
3‐stage exchange arthroplasty	18 (16.1%)	10 (38.5%)	0.043*
4‐stage exchange arthroplasty	2 (1.8%)	2 (7.7%)	0.012*
History of subsequent PJI < 3 months interval	7 (6.23%)	6 (26.1%)	0.024*
Bacteria of first DAIR			
Culture‐negative	40 (35.7%)	9 (34.6%)	n.s.
Gram‐positive	55 (49.1%)	12 (46.2%)	n.s.
Gram‐negative	14 (12.5%)	4 (15.4%)	n.s.
Fungus	4 (3.6%)	1 (3.8%)	n.s.
Tuberculosis	3 (2.7%)	0 (0%)	n.s.
Poly‐microbial	22 (19.6%)	4 (15.4%)	n.s.
Enterococcus	0 (0%)	0 (0%)	n.s.
MRSA	19 (17.0%)	4 (15.4%)	n.s.
Bacteria of second DAIR			
Culture‐negative	N/A	12 (46.2%)	
Gram‐positive	N/A	8 (30.8%)	
Gram‐negative	N/A	4 (15.4%)	
Fungus	N/A	2 (7.7%)	
Tuberculosis	N/A	0 (0%)	
Poly‐microbial	N/A	4 (15.4%)	
Enterococcus	N/A	0 (0%)	
MRSA	N/A	4 (15.4%)	
Same species of bacteria on last DAIR and staged exchange arthroplasty	27 (24.1%)	8 (30.8%)	0.032*
Same species of bacteria on first and second DAIRs	N/A	10 (38.5%)	

Abbreviations: CI, confidence interval; DAIR, surgical debridement, antibiotics and implant retention; MRSA, methicillin‐resistant *Staphylococcus aureus*; N/A, not available; PJI, periprosthetic joint infection.

* *p* value < 0.05.

### Duration of treatment and follow‐up

The follow‐up duration was similar between the groups (one DAIR before EA and second DAIR before EA) (*p* = n.s.). However, the time from the first DAIR to two‐stage EA was significantly shorter in the one DAIR group compared to the second DAIR group (502.1 days vs. 803.2 days; *p* = 0.041). The interval between the first and final stage of EA was also shorter in the one DAIR group (126.6 days vs. 170.9 days; *p* = 0.046) (Table 3).

**Table 3:** Timetable differences of patients who underwent staged exchange arthroplasty after first or second DAIR.

Variables	First DAIR (112)	Unplanned second DAIR (26)	*p*
Timetable of DAIR (days)			
Primary TKA to first DAIR (median) (range) (SD)	675.12 (321) (234–1203) (123.42)	703.2 (401) (153–1134) (178.27)	n.s
First stage from first DAIR (median) (range) (SD)	502.11 (423.1) (392–1229) (273.26)	803.21 (834.5) (792–1376) (229.14)	0.041*
First stage from second DAIR (median) (range) (SD)	N/A	396.37 (388.2) (384–1350) (214.64)	X
Time from first DAIR to second DAIR (median) (range) (SD)	N/A	810.33 (754) (754–1241) (174.05)	X
Follow‐up (median) (range) (SD)	3767.3 (3906.5) (1719–5736) (1204.4)	3921.38 (4145) (755–6156) (1445.36)	n.s.
Interim between exchange arthroplasty (median) (range) (SD)	126.58 (94.5) (98–873) (103.99)	170.85 (98) (99–1078) (232.78)	0.046*

Abbreviations: DAIR, surgical debridement, antibiotics and implant retention; N/A, not available; SD, standard deviation; TKA, total knee arthroplasty.

* *p* value < 0.05.

### Multivariate analysis of infection‐free survivorship and implant failure

**Figure 2:**
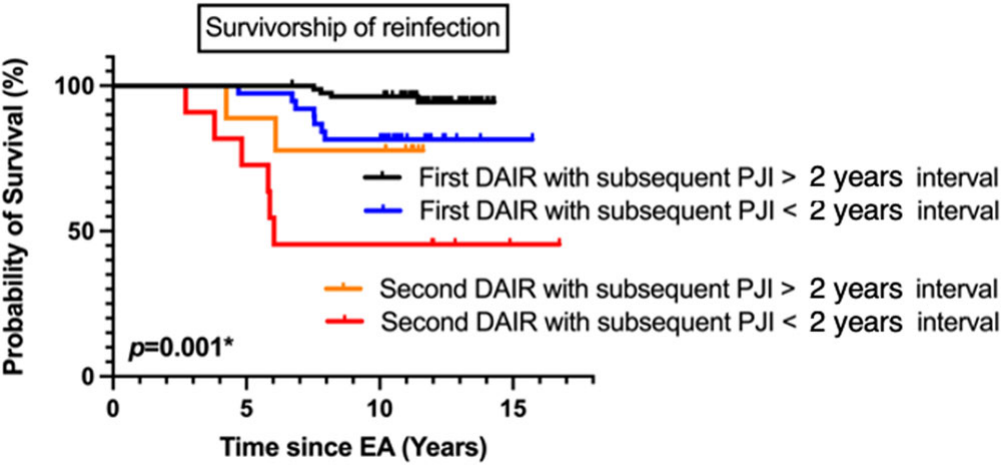
Survival curve of staged exchange arthroplasty free from reinfection by times of debridement, antibiotics and implant retention (DAIR). Follow‐up time was defined as the time from the final staged EA (reimplant date for staged EA) to the date of reinfection, death or study end date (31 December 2020), whichever came first. Censored data (vertical spikes) revealed reinfection. Death was treated as a competing event, while reinfection and study end date were censoring events. Groups: ‘First DAIR with subsequent PJI > 2 years interval’; ‘First DAIR with subsequent PJI < 2 years interval’; ‘Second DAIR with subsequent PJI > 2 years interval’; ‘Second DAIR with subsequent PJI < 2 years interval’; EA, exchange arthroplasty; PJI, periprosthetic joint infection; *p value < 0.05.

**Figure 3:**
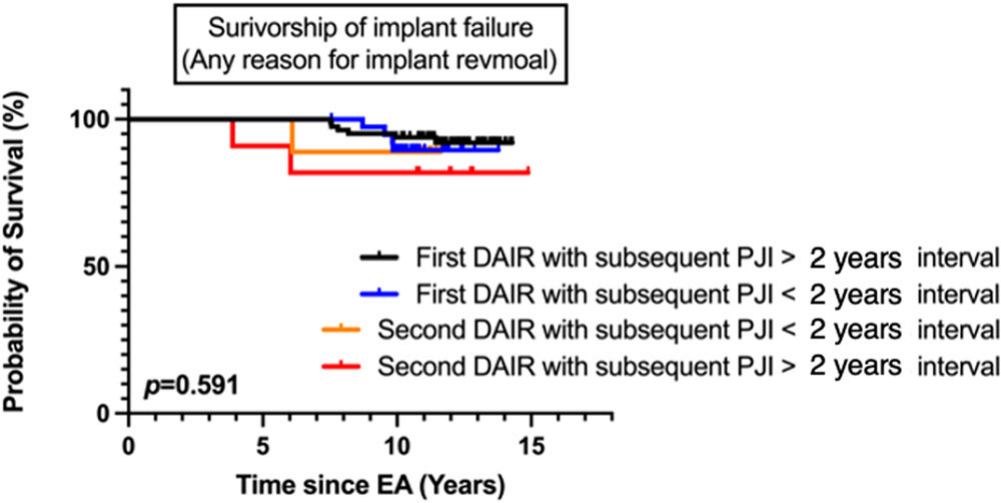
Survival curve of staged exchange arthroplasty free from revision by times of debridement, antibiotics and implant retention (DAIR). Follow‐up time was defined as the time from the final staged EA (reimplant date for staged EA) to the date of revision surgery, death, or study end date (31 December 2020), whichever came first. Censored data (vertical spikes) revealed revision surgery. Death was treated as a competing event, while revision and study end date were censoring events. Groups: ‘First DAIR with subsequent PJI > 2 years interval’; ‘First DAIR with subsequent PJI < 2 years interval’; ‘Second DAIR with subsequent PJI > 2 years interval’; ‘Second DAIR with subsequent PJI < 2 years interval’. EA, exchange arthroplasty; PJI, periprosthetic joint infection. *p value < 0.05.

Infection‐free survivorship was significantly lower in patients who underwent second DAIR(s) prior to two‐stage EA with a history of subsequent PJI within a 2‐year interval (*p* = 0.002). However, the survivorship of implants did not show a significant difference (*p* = n.s.) (Figures 2 and 3). The risk of reinfection was higher in cases with pre‐first DAIR CRP levels >100 mg/L (HR = 2.52; 95% CI = 1.98–3.42; *p* = 0.003), patients undergoing unplanned second DAIR before two‐stage EA with a history of subsequent PJI in <2 years (HR = 4.23; 95% CI = 2.39–5.31; *p* = 0.002), or with the same pathogen recurring between the last DAIR and EA (HR = 2.35; 95% CI = 1.32–4.24; *p* = 0.007). A higher risk of implant failure was observed in males (HR = 3.92; 95% CI = 1.21–5.25; *p* = 0.007) and patients with osteoporosis (T score < −2.5; HR = 3.27; 95% CI = 1.23–5.28; *p* = 0.005) (Table 4). The risk factor for unplanned second DAIR failure was identified as the recurrence of the same pathogen from the first DAIR (HR = 3.41; 95% CI = 1.35–4.38; *p* = 0.004) (Table 5).

**Table 4:** Results of multivariate Cox regression analysis of variables associated with treatment failure (reinfection or implant failure) (hazard ratio [95% CI]).

	Multivariate model results	
Variables	Hazard ratio (95% CI)	*p*
Reinfection (any episode of PJI)		
CRP level > 100 mg/dL before first DAIR	2.52 (1.98–3.42)	0.003*
Second DAIRs before staged EA with history of subsequent PJI > 2 years interval	2.65 (0.19–3.19)	n.s.
Second DAIRs before staged EA with history of subsequent PJI < 2 years interval	4.23 (2.39–5.31)	0.002*
The same pathogen between last DAIR and EA	2.35 (1.32–4.24)	0.007*
Interim between exchange arthroplasty procedures > 120 days	0.33 (0.11–2.12)	n.s.
Ever treated with repeat unplanned second DAIR	0.12 (0.13–2.87)	n.s.
3‐stage EA	0.42 (0.23–1.87)	n.s.
4‐stage EA	0.33 (0.21–2.86)	n.s.
Implant failure (any reason to remove implant)		
Male	3.92 (1.21–5.25)	0.007*
Osteoporosis with T score < −2.5	3.27 (1.23–5.28)	0.005*
Second DAIRs before staged EA with history of subsequent PJI > 2 years interval	2.88 (0.12–4.24)	n.s.
Second DAIRs before staged EA with history of subsequent PJI < 2 years interval	2.43 (0.29–3.44)	n.s.
Interim between exchange arthroplasties > 120 days	1.47 (0.12–2.63)	n.s.
Ever treated with repeat unplanned second DAIR	1.64 (0.24–1.28)	n.s.
3‐stage EA	1.44 (0.14–1.98)	n.s.
4‐stage EA	1.34 (0.26–3.31)	n.s.

Abbreviations: CI, confidence interval; CRP, C‐reactive protein; DAIR, surgical debridement, antibiotics and implant retention; EA, exchange arthroplasty; PJI, periprosthetic joint infection.

* *p* value < 0.05.

**Table 5:** Results of multivariate Cox regression analysis of variables associated with treatment failure of second DAIR (reinfection) (hazard ratio [95% CI]).

	Multivariate model results	
Variables	Hazard ratio (95% CI)	*p*
Reinfection (any episode of PJI)		
The same pathogen as first DAIR	3.41 (1.35–4.38)	0.004*
Different species of bacteria on first and second DAIR	2.92 (0.17–3.12)	n.s.
Improvement of CRP level but fluctuated during 6‐week antibiotic treatment	1.89 (0.31–3.13)	n.s.
Gradually normalized CRP level during 6‐week antibiotic treatment	1.28 (0.03–1.97)	n.s.
Subsequent PJI < 2 years interval after first DAIR	1.41 (0.41–5.31)	n.s.
Subsequent PJI > 2 years interval after first DAIR	2.43 (0.29–3.44)	n.s.

Abbreviations: CI, confidence interval; DAIR, surgical debridement, antibiotics and implant retention; EA, exchange arthroplasty; PJI, periprosthetic joint infection.

* *p* value < 0.05.

## DISCUSSION

The most important finding of this study was the determination of the success rates and risk factors associated with unplanned second DAIRs and two‐stage EA following PJI with a success rate of only 80.6% of unplanned second DAIR. The major risk factor for failure was the same pathogen as previous DAIR. Comparatively, the overall success rate for EA after DAIR was 87.0%, with a notably higher success rate after the one DAIR (90.2%) than after an unplanned second DAIR (69.2%).

Growing evidence reveals that DAIR could provide comparable infection control and better bone stock preservation without the need to remove the prosthesis [13]. Regarding reinfection after DAIR, the literature shows no compromise in success rates when following a two‐stage EA according to a strict protocol [17].

At present, an increasing number of surgeons are performing multiple DAIRs on cases of recurrent acute PJI. A planned second DAIR was performed on some studies [6], which differs from the approach used in ours. For unplanned second DAIR, Wouthuyzen‐Bakker et al. studied patients receiving two DAIRs for hip and knee arthroplasty. The second DAIR had a low failure rate (37/144, 25.7%), with most implants retained (107/144). Predictors of failure included positive cultures during the second DAIR and chronic renal insufficiency [34]. However, Wouthuyzen‐Bakker et al. excluded patients with unplanned second DAIR after a 3‐month interval of the first DAIR and did not find any significant difference in failure risk between persistent infection and new infection. Our patients received unplanned second DAIR for acute knee PJI following our treatment protocol after claiming the successful first DAIR longer than 1 year within a mean interval of 810.33 days and achieved an 80.57% success rate. Even though, in our cohort, some patients with unplanned second DAIR might be due to the same pathogen as previous DAIR or new infection, or even both. In each DAIR procedure, we exchanged the modular component whenever possible to minimize the risks of persistent infection, and to broaden the field of debridement [32]. However, 30% of our patients were cultured with the same pathogen between DAIRs. A higher failure rate for unplanned second DAIR was related to persistent infection following the first DAIR, which was comparable with previous studies [15, 28].

Moreover, this phenomenon of persistent infection was also observed in the subsequent two‐stage EA in our cohort. The same pathogen between the last DAIR and index EA was also found to have increased reinfection risk in our regression analysis (HR = 2.35, 95% CI = 1.32–4.24) for the subsequent two‐stage EA. Persistent infection could explain why the unplanned second DAIR group suffered from “more‐stages” EA in our cohort. This might facilitate the pathogen becoming a more resistant one which could benefit from spacer exchange to the identified resistant pathogen [7].

Despite the culture‐negative rate (35.7% to 34.6% in the first DAIR vs. 46.2% in the unplanned second DAIR), our study was unable to definitively discern whether persistent infection, new infection, or a combination of both were present. Modern techniques with next‐generation sequencing [11] and machine‐learning model might be helpful in the future [18]. This limitation led us to implement a time interval of 2 years after the completion of antibiotic treatment to evaluate if a subsequent PJI occurred. Our findings indicated that the occurrence of a PJI within 2‐year post‐treatment is associated with a reduced survivorship for EA following a second DAIR. It appears that the occurrence of a subsequent PJI within 2 years plays a pivotal role in differentiating survivorships for the subsequent EA.

A recent review article concluded that the lower the CRP level, the better outcomes could be achieved with DAIR [21, 26, 35, 37]. Our study observed a similar trend. Patients with pre‐first DAIR CRP levels >100 mg/L had 2.52‐fold higher hazard of reinfection after two‐stage EA.

Risk of implant failure was significantly higher in male patients and in osteoporotic cohorts, with hazard ratios of 3.92 and 3.27, respectively. Ferry et al. proposed that DAIR in knee joints might lead to a higher implant failure rate despite the absence of soft tissue impairment [9]. A higher risk of PJI has been illustrated in the male population in previous studies [36]. Johnsen et al. revealed male sex was associated with higher implant failure rate probably related to higher physical demands and body weight [16]. Severe osteoporosis (T score <−2.5) increases implant failure rates, potentially due to periprosthetic fractures. It is a significant risk factor for surgery‐related complications, including aseptic loosening or periprosthetic fractures.

There were various limitations to the study. To begin with, we did not compare the rate of treatment failure between a second DAIR and staged EA after an initial DAIR procedure. The literature has conflicting results regarding the outcome of these patients [5, 10, 22, 25] and our study could not answer the question due to the possible selection bias from the retrospective nature. Second, during long‐term follow‐up, the possibility of undetected PJI may arise because it did not meet the criteria outlined in our protocol. To reduce the risk of undetected PJI, we have incorporated cultures for slow‐growing organisms like Cutibacterium or Mycobacterium. Third, the small sample size could result in a type II error, especially for multiple comparisons. As a result, we have performed multiple comparison tests to minimize the error.

## CONCLUSIONS

Conclusively, our research highlights the paramount risk factors leading to suboptimal outcomes in EA post‐DAIR, notably a pre‐first DAIR CRP level over 100 mg/L, the reemergence of the identical pathogen, and a preceding PJI occurrence within a two‐year frame. The evidence underscores the necessity of prudent assessment before proceeding with an unplanned second DAIR, reflecting its significant influence on the results of ensuing EA. Moreover, our analysis reveals that the probability of implant failure remains consistent across all EA cases, irrespective of a preceding first or second DAIR, particularly among patients exhibiting risk factors such as male gender and severe osteoporosis (T score < −2.5).

## AUTHOR CONTRIBUTIONS

Yu‐Chih Lin participated in the design of the study and writing of the final manuscript. Wei‐Cheng Chen participated in the sequence alignment. Yu‐Chih Lin and Shih‐Hui Peng performed the statistical analysis. Chih‐Hsiang Chang, Sheng‐Hsun Lee and Sheng‐Hsuan Lin all contributed to the design of the article. Yu‐Chih Lin conceived the study, participated in its design and coordination, and helped assemble the manuscript draft. All authors read and approved the final manuscript.

## CONFLICT OF INTEREST STATEMENT

The authors declare no conflict of interest.

## ETHICS STATEMENT

Our study involved the comparison and analysis of de‐identified, population‐level data. Our study was approved by the Institutional Review Board of our hospital (IRB: 201601034B0). The study was performed at Chang Gung Memorial Hospital (CGMH). Informed consent was waived due to the retrospective nature of the study. The authors affirm that human research participants provided informed consent for publication of the images in Figure 1 and Tables 1, 2, 3.

## Data Availability

The data, materials and/or code generated during and/or analysed during the current study are available from the corresponding author on reasonable request.
